# Cataract risk stratification and prioritisation protocol in the COVID-19 era

**DOI:** 10.1186/s12913-021-06165-1

**Published:** 2021-02-17

**Authors:** Kelvin KW Cheng, Martin J Anderson, Stavros Velissaris, Robert Moreton, Ahmed Al-Mansour, Roshini Sanders, Shona Sutherland, Peter Wilson, Andrew Blaikie

**Affiliations:** 1 Ophthalmology Department, NHS Fife, Queen Margaret Hospital, Fife Dunfermline, UK; 2 University of Edinburgh, Edinburgh, UK; 3 University of St Andrews, St. Andrews, UK

## Abstract

**Background:**

The COVID-19 pandemic halted non-emergency surgery across Scotland. Measures to mitigate the risks of transmitting COVID-19 are creating significant challenges to restarting all surgical services safely. We describe the development of a risk stratification tool to prioritise patients for cataract surgery taking account both specific risk factors for poor outcome from COVID-19 infection as well as surgical ‘need’. In addition we report the demographics and comorbidities of patients on our waiting list.

**Methods:**

A prospective case review of electronic records was performed. A risk stratification tool was developed based on review of available literature on systemic risk factors for poor outcome from COVID-19 infection as well as a surgical ‘need’ score. Scores derived from the tool were used to generate 6 risk profile groups to allow prioritised allocation of surgery.

**Results:**

There were 744 patients awaiting cataract surgery of which 66 (8.9 %) patients were ‘shielding’. One hundred and thirty-two (19.5 %) patients had no systemic comorbidities, 218 (32.1 %) patients had 1 relevant systemic comorbidity and 316 (46.5 %) patients had 2 or more comorbidities. Five hundred and ninety patients (88.7 %) did not have significant ocular comorbidities. Using the risk stratification tool, 171 (23 %) patients were allocated in the highest 3 priority stages. Given an aging cohort with associated increase in number of systemic comorbidities, the majority of patients were in the lower priority stages 4 to 6.

**Conclusions:**

COVID-19 has created an urgent challenge to deal safely with cataract surgery waiting lists. This has driven the need for a prompt and pragmatic change to the way we assess risks and benefits of a previously regarded as low-risk intervention. This is further complicated by the majority of patients awaiting cataract surgery being elderly with comorbidities and at higher risk of mortality related to COVID-19. We present a pragmatic method of risk stratifying patients on waiting lists, blending an evidence-based objective assessment of risk and patient need combined with an element of shared decision-making. This has facilitated safe and successful restarting of our cataract service.

## Background

Cataract is the most common cause of treatable visual impairment in elderly patients in the UK accounting for over a third of visual impairment in those over 75 years [1]. Due to its recognised cost and clinical effectiveness, it is one of the most common surgical interventions performed in wealthy nations [2–5]. Visual impairment and age-related cataract is known to be associated with increased mortality [6–9]. In addition, visual impairment is also associated with increased risk of depression after adjusting for confounding factors [10]. Improved vision reduces the risk of falls and hip fractures as well as of developing dementia [11]. Improved quality of life with improved independence with less need for hospital and community care makes cataract surgery service restart a priority for NHS Scotland in the COVID-19 era [12–15].

Several studies show that COVID-19 disproportionately affects the elderly, those with multiple comorbidities [16] and people from Black, Asian and Minority Ethnic (BAME) groups. As cataract prevalence increases with age and may be accelerated by comorbidities, the demographic benefiting from cataract surgery is also at high risk of poor outcome from COVID-19 infection. This poses significant practical challenges of safely restarting services and alters the balance of risk and benefit of surgery to also include the adverse outcomes of COVID-19 infection.

The Royal College of Ophthalmologists (RCO) and United Kingdom and Ireland Society of Cataract and Refractive Surgery (UKISCRS) have recently provided guidelines on recommencing cataract surgery services post-lockdown [17]. Taking the guidelines, systemic risk factors for poor outcome from COVID-19 and patient visual needs into consideration, we developed and implemented a risk stratification tool and prioritisation system relevant to cataract surgery in the COVID-19 era. The tool takes into account individual risk of morbidity and mortality associated with COVID-19 infection, alongside separate considerations regarding the risks and benefits of cataract surgery itself. The patient journey from home to hospital, as well as time spent in hospital during surgery increases risk of exposure to COVID-19 and therefore warrants consideration in the consent process for cataract surgery.

**Fig. 1:**
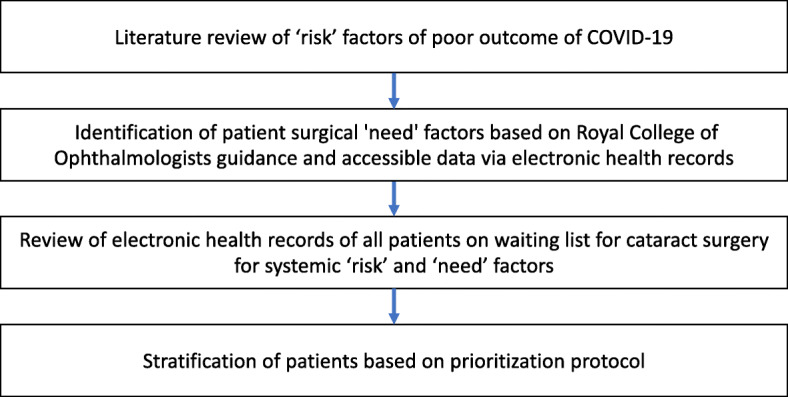
Flowchart of research methodology

A recent qualitative study from the United Kingdom reported that over 80 % of patients were willing to undergo cataract surgery despite the risks of contracting COVID-19 while travelling in the community and attending hospital. Younger age, male sex and the need to drive [18] were the main characteristics of patients who were most motivated to prioritise improvement of vision over the potential morbidity from COVID-19 infection. These patients however may not be the ones most in need of surgery. With high and often unpredictable demands on health services managing COVID-19 admissions, any restart and growth of elective cataract activity should make every attempt to maximise the health benefits from intervention while minimising risks of adding to the burden of COVID-19 admissions. This paper describes the process of development of a tool that deals with this tension between desire for surgery, risk of a poor outcome of contracting COVID-19 and the actual need for surgery. In addition, we report the demographics and comorbidities of all patients waiting for cataract surgery in NHS Fife and the stratification groups of patients generated according to the prioritisation protocol. Figure 1 illustrates the process involved in the development of this stratification and prioritisation protocol.

## Methods

 We performed a prospective review of all patients on the cataract surgery waiting list in collaboration with the NHS Fife Quality Improvement Register. Using the NHS Clinical Portal age, sex, ethnicity and systemic comorbidities (Table 1), visual acuity, degree of anisometropia and presence of other ocular disease were recorded. Patients were assigned a systemic ‘risk’ score based on their likelihood of poor outcome from COVID-19 infection and a separate surgical ‘need’ score based on the risks and benefits of cataract surgery itself, encompassing level of visual acuity, degree of anisometropia, presence of only one good eye and co-existent ocular disease that might limit outcome from surgery (Table 1). Patients who were known to be ‘shielding’ were excluded from detailed data analysis as they did not require further risk stratification. They were already deemed to be of highest risk of poor outcome from COVID-19 infection due to serious underlying systemic illness.

**Table 1:** ‘Risk’ and ‘Need’ Score For Cataract Surgery Prioritisation

Characteristic	Score
**Risk Score (Based on systemic risk to poor COVID-19 outcome)**	
**Age**	
< 70 years	0
70 to 79 years	5
> 80 years	10
**Sex** (Male)	1
**Ethnicity** (BAME)	1
**Systemic Comorbidities**	
Chronic respiratory disease (excluding mild asthma)	1
Chronic heart disease (excluding hypertension)	1
Chronic Kidney Disease	1
Malignancy	1
Diabetes	1
Other	1
**Maximum Risk score**	**18**
**Need Score (Based on need and prognosis of cataract surgery)**	
Vision 6/12 or worse OR anisometropia > 3D	1
Lack of any of the following ocular comorbidities significantly affecting prognosis of surgery	1
Moderate to Advanced AMD	
Amblyopia	
Cornea opacification	
Retinal artery/vein occlusion	
Chronic retinal degeneration	
Central visual impairment	
Optic neuropathy	
Active uveitis	
Other maculopathy (excluding AMD)	
Only one good eye	1
**Maximum Need Score**	**3**

### Cataract surgery prioritisation stages

**Fig. 2:**
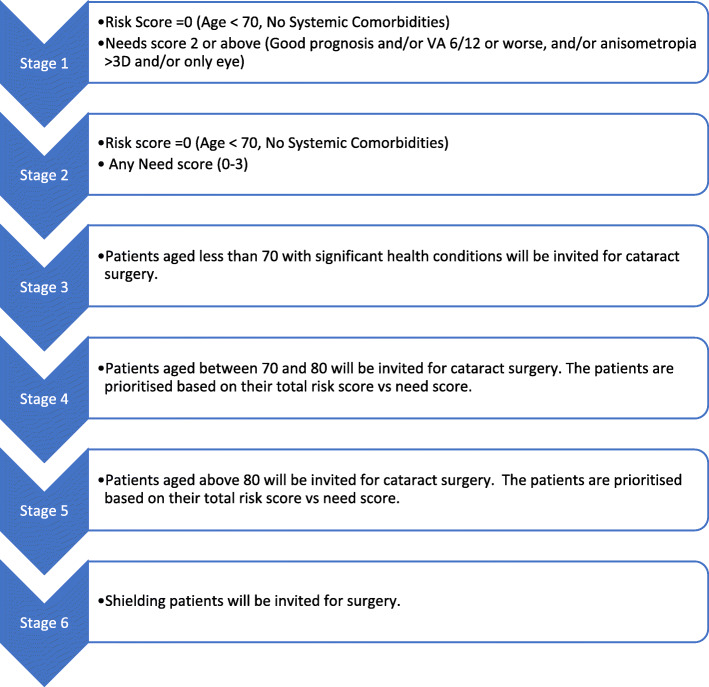
Proposed Cataract Surgery Prioritisation Stages based on patients’ risk of poor outcome from COVID-19 and ‘need’ for surgery

We stratified patients on the waiting list based on a balance between their ‘risk’ and ‘need’ prioritising those with the greatest ‘need’ and lowest ‘risk’. This led to the creation of 6 different phased stages (Fig. 2). In each stage, patients with the greatest need score and lowest risk score are prioritised. Patients with a vision 6/12 or worse, or anisometropia of > 3D without systemic and ocular comorbidities are invited first in Stage (1) Patients with vision of better than 6/12 with or without known ocular comorbidities without systemic comorbidities are invited in stage (2) Patients of more advanced age or with comorbidities are invited for surgery at a later point in stages 3,4 and 5, as described in Fig. 2. Patients that were ‘shielding’ were advised that their routine surgery was postponed until new guidance is issued by the Scottish Government [19] given that ‘shielding’ patients were deemed to be of highest risk of poor outcome from COVID-19 infection due to serious underlying systemic illness. Patients within each stage are prioritised based on first ‘risk’ then ‘need’ score, as systemic risk related to COVID-19 was deemed to take priority. Further, the patient-led recall system allowed patients to express their wish for surgery despite increased risk of mortality from a potential COVID-19 infection and these patients were prioritised within their stage.

To illustrate this, we will model the process for three fictional patients. Patient A is 67-year-old female with no systemic co-morbidities, yielding a risk score of zero, suitable for stages 1 and 2. However, the patient’s vision is 6/9 in both eyes at the time of initial assessment, with no ocular co-morbidities yielding a ‘need’ score of 1 thus qualifying the patient for stage 2. Patient B is a 75-year-old male with a history of chronic heart disease but no ocular co-morbidities and vision of 6/12, yielding a ‘risk’ score of 7 and ‘need’ score of 2, thus qualifying for stage 4 based on age. Patient C is a 73-year-old male with no systemic or ocular co-morbidities and vision of 6/18, yielding a ‘risk’ score of 6 and ‘need’ score of 2. Patient C will be allocated to stage 4 based on age but will be prioritised within stage 4 over Patient B due to a lower ‘risk’ score.

The patients were then sent a letter to explain the likely timing of their surgery. Patients were offered the opportunity to telephone the department if they wished to raise any concerns regarding unrecognised need for improved vision or significant progression of sight loss since original referral. A phone consultation with the operating consultant was then offered to clarify their wish to have surgery after further discussion of their individual potential risk of contracting COVID-19 during their hospital visit in accordance with the RCO’s guidance.

### Statistical analysis

Median and interquartile range (IQR) were used to summarise continuous results after inspection of results using histograms. Student T-test was used to measure any differences in number of comorbidities between patients aged below and above 70. Linear regression was used to measure the relationship between age and number of systemic comorbidities. P-values of < 0.05 were considered statistically significant. Data was analysed using R software (3.5.1, R Foundation for Statistical Computing, AUT, Vienna, Austria).

## Results

### Demographics

Table 2 describes the demographics of patients awaiting cataract surgery. Five hundred and ninety patients (88.7 %) did not have any ocular comorbidities. Moderate to advanced age-related macular degeneration was the most common ocular comorbidity affecting 31 patients (4.7 %) followed by amblyopia affecting 11 patients (1.7 %) and macular pathology of any cause (excluding AMD) affecting 10 patients (1.5 %).

### Comorbidities and risk score

**Fig. 3:**
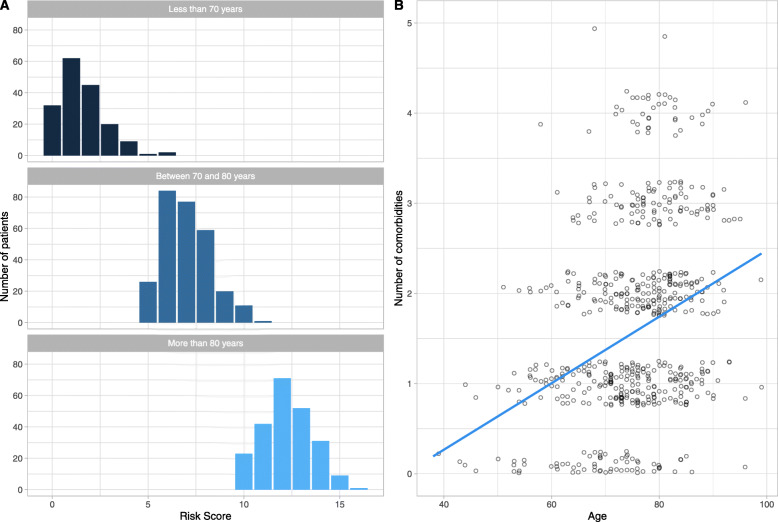
**a**. Risk Score of patients in each age group, **b.** The number of comorbidities increased significantly with age

The frequency of comorbidities recorded are listed in Table 2. One hundred and thirty-two (19.5 %) patients had no comorbidities, 218 (32.1 %) patients had only 1 comorbidity and 316 (46.5 %) patients were identified to have 2 or more comorbidities. Figure 3A illustrates the range of risk scores in this cohort of patients. The risk score has a median of 7 (IQR 5 to 11). Of the 508 patients aged 70 and above, only 70 (11.4 %) patients did not have any systemic comorbidities. With every year increase in age, the number of comorbidities increases by 0.037 (R^2^ = 0.093, P < 0.001) as seen in Fig. 3B. There was a significant difference in the number of systemic comorbidities between patients aged below (1.08) and above 70 (1.74) (P < 0.001, CI = -0.85 to -0.48).

**Table 2:** Demographics and frequencies of ocular and systemic comorbidities. Data presented in Median (IQR) unless specified otherwise

Demographics	Results
Sex (% Females)	393 (55.4)
Ethnicity (% Whites)	628 (98.4)
Age	76 (69 to 82)
Waiting List Duration in weeks	6.8 (3.9 to 9.6)
**Ocular Comorbidities**	**n (%)**
Moderate to severe AMD	31 (4.7)
Amblyopia	11 (1.7)
Cornea opacification	6 (0.9)
Retinal artery/vein occlusion	6 (0.9)
Chronic retinal degeneration	4 (0.6)
Central visual impairment	4 (0.6)
Optic neuropathy	2 (0.3)
Active uveitis	1 (0.2)
Other maculopathy (excluding AMD)	10 (1.5)
**Systemic Comorbidities**	
Chronic heart disease	194 (28.9)
Diabetes	148 (22.1)
Chronic kidney disease	139 (20.7)
Chronic respiratory disease	66 (9.9)
Malignancy	39 (5.8)
Others	458 (68.3)

### Cataract surgery prioritisation stages

Table 3 illustrates the number of patients allocated to each stage of resumption outlined in Figure 2. The majority of patients were allocated to stages 4 to 6 (573, 77.1%) due to a large proportion of patients being of advanced age with at least one systemic comorbidity.

**Table 3:** Number of patients in each cataract surgery prioritisation stage

Stages	Number of patients (%)
1	28 (3.8)
2	37 (5.0)
3	106 (14.2)
4	278 (37.4)
5	229 (30.8)
6	66 (8.9)

### Patient‐led recall

Seven hundred forty-four patient letters, containing basic information on prioritisation stage, surgery-related COVID-19 risk and surgical prognosis were sent. To date (5 weeks since patient letters were sent), 53 (7.1 %) patients have telephoned the department requesting a discussion with their consultant regarding their allocated priority stage. We are not aware of any difficulties faced by patients contacting the department and most telephone calls were received within 3 weeks of patients receiving their allocation letters.

## Discussion

The coronavirus pandemic has led to significant disruption of routine cataract surgery in the UK and will continue to impact capacity in unpredictable ways for the foreseeable future. This situation requires all ophthalmic departments to develop a plan for safe surgical prioritisation. A combination of increased waiting lists due to disruption of services and reduced capacity going forward pose challenges that must be addressed to deliver services safely.

The RCO guidance on resumption of cataract surgery highlighted the need to balance quality of life, surgical risk and the risk to patients of COVID-19 infection [17]. The risk stratification and patient prioritisation protocol we describe was designed with several aims; (1) quantify the risk factors that were known at the time to be predictors of poor outcomes in patients with COVID-19 infection in our patients waiting for cataract surgery, (2) categorise patients by need and from this, (3) derive an approach to cataract surgery resumption that is efficient and pragmatic [20]. Available peri-operative risk prediction tools such as the Acute Physiology and Chronic Health Evaluation (APACHE II) and Physiological and Operative Severity Score for the enumeration of Mortality and Morbidity (POSSUM) scores are dependent on physiological variables, such as heart rate, blood pressure and laboratory values. These are more relevant to a surgical procedure requiring a general anaesthetic and not a local anaesthetic cataract operation. In addition up to date physiological variables were not readily available to us. Further, these scoring systems do not take into account risk factors specific to COVID-19, such as ethnicity and sex [21, 22].

Many studies have shown that age is the single biggest predictor of poor outcome in COVID-19 patients with a large increase in risk in patients aged 70 and above. Docherty et al. reported that after adjusting for major comorbidities, age between 70 and 79 was associated with a hazard ratio of 8.51 while patients aged 80 and above were associated with a hazard ratio of 11.09, compared to patients less than 50 years, in terms of mortality [16]. The results of this study concurs with the OpenSAFELY study which employed a secure health analytics platform that covers 40 % of all patients in England which pseudonymously linked the primary care records of over 17 million adults to COVID-19 deaths. In this study, ages 70 to 79 was associated with an adjusted COVID-19 death hazard ratio of 6.07 (5.51 to 6.69) and ages 80 and above with a hazard ratio of 20.60 (18.70 to 22.68) [23]. We felt that given age is the single biggest predictor of mortality in COVID-19, age should be given more weighting in any prioritisation score and guide discussion with patients during the consent process. Therefore, a score of 5 points for ages 70 to 80 and 10 for ages more than 80 reflects the importance of age in our risk stratification model. The age intervals were chosen based on available risk and hazard ratios reported by studies. Studies also reported an increase in morbidity in males and in individuals of BAME background and therefore ethnicity and sex were also accounted for in our scoring system [16, 23]. The prioritisation stages we developed based on systemic ‘risk’ and ‘need’ demonstrate that a significant number of patients awaiting cataract surgery are, as expected, of high risk of poor outcome from COVID-19 and therefore every effort to reduce the risk of these patients contracting COVID-19 should be considered and used to inform prioritisation for surgery.

Identification of the most important comorbidities that are predictors of mortality in COVID-19 formed the basis of selection of comorbidities for risk stratification. At the time of development of our risk scoring system, there were several reports from China and only 2 from the UK [16, 23–26]. Of the comorbidities studied, chronic heart, respiratory and kidney disease, malignancy and diabetes stood out as important risk factors and therefore were specifically recorded. The hazard ratios for each comorbidity varied significantly between studies and therefore the additional score of 1 per known associated risk factor was felt to be practical. Importantly, recording which comorbidity was scored allows for retrospective weighting to be carried out if future evidence on COVID-19 risk highlights particularly high-risk comorbidities or demographic characteristics.

We acknowledge that a binary grading of visual acuity using a cut off of 6/12 lacks granularity and fails take into account the gradual increase in risk of falls and impaired quality of life as vision deteriorates. Visual acuity of worse than 6/12 was however felt to be a worthwhile point to choose as driving would no longer be legal or safe and reading vision significantly impaired [27]. This simple approach offered a swift and pragmatic means to process all the patients on the waiting list and the compromise of this strategy was accepted to ensure we completed the task. Although visual acuity is strongly associated with cataract severity, visual acuity measurement does not take into account glare and contrast sensitivity affecting quality of life. In addition, glare disability with cataracts does not correlate with visual acuity [28, 29]. However, given the volume of information needed for even a simplified prioritisation model alongside the large number of patients on the waiting list, a detailed discussion regarding specific visual symptoms and quality of life factors would significantly increase the workload associated with patient prioritisation. In our model, the patient-led opportunity to discuss their priority group was deemed an acceptable way for them to discuss and express their wishes for surgery based on their own perception of risk of poor outcome from COVID-19 infection and their desire for pursing surgery to improve their vision. The low number of patients that got in touch we believe reflects the appropriateness of our prioritisation tool.

The prioritisation plan was designed with the aim of balancing risk of mortality secondary to COVID-19 infection and potential benefits of cataract surgery. Patients with the lowest risk and most potential gain from cataract surgery were invited first. Although the presence of ocular comorbidities does not preclude the potential of improvement in visual acuity post-surgery, it is difficult to anticipate the level of expected improvement, especially without further face to face patient review. The Blue Mountains Eye five-year follow-up study reported that early age-related maculopathy at baseline adversely affected the postoperative visual acuity following cataract surgery [5]. Armbrecht et al. reported that patients with no ocular comorbidities had more pronounced improvements in quality of life measures and visual function post-operatively [30]. Given that age is the most important predictor of mortality in COVID-19, these patients are only invited in the later stages of the plan. It is hoped that this will provide more time for the pandemic to settle, hospital procedures to be optimised to reduce risk, and thus be safer for these patients to attend for their surgery. However, given the patient-led recall aspect of our model, all patients have the opportunity to discuss being prioritised for surgery regardless of their risk score. In this study, most patients were stratified into stages 4 to 6. The resulting small number of patients in Stages 1 to 3 led to these patients being invited for cataract surgery promptly.

We note several limitations to our stratification system and prioritisation model. As we learn more about risk factors affecting mortality associated with COVID-19, the selected comorbidities for scoring may need to be expanded and given a wider range of weightings. In our study, a significant proportion of patients were noted to have hypertension which was recorded under ‘Other’ but in retrospect, this could be recorded as a separate condition in future models [31]. The electronic health data available for patients may be outdated by several months given the reduction in contact with the health service during the COVID-19 pandemic. The reported visual acuity in the initial referral and assessment may have deteriorated significantly while awaiting cataract surgery. The patient-led recall model is a pragmatic approach to take this into account and also allows patients to highlight the negative impact upon their activities of daily living which they would also like to be taken into consideration. Our cohort is not diverse with only 1.6 % being of BAME ethnicity. Therefore, extrapolation to more ethnically diverse populations maybe limited.

## Conclusions

In conclusion, we believe that our pragmatic tool provides a simple, quick and effective means to prioritise patients waiting for cataract surgery that takes into account their risks of poor outcome from COVID-19 infection and need for improvement in vision from surgery. This approach we believe successfully balances the risk of morbidity and mortality from COVID-19 infection, need for improved vision and the potential of visual improvement from surgery. The demographic and comorbidity data shows, as we expected, that the majority of our patients are at an increased risk of poor outcome from COVID-19 infection. This new concern drives the need for a change in our assessment of risks and benefits of an operation that was previously considered a low-risk intervention. Given the growing knowledge of COVID-19, it is anticipated that the systemic risk factors selected and their relative weighting may need to be modified in the future. We believe however that this tool provides a simple and pragmatic starting point for developing stratification and prioritisation protocols which can be modified as the pandemic evolves.

## Data Availability

The datasets used during the current study are available from the corresponding author on reasonable request.

## Abbreviations

AMD: Age-related Macular Degeneration
APACHE II: Acute Physiology and Chronic Health Evaluation
BAME: Black, Asian and Minority Ethnic
NHS: National Health Service
POSSUM: Physiological and Operative Severity Score for the enumeration of Mortality and Morbidity
RCO: Royal College of Ophthalmologists
UKISCRS: United Kingdom and Ireland Society of Cataract and Refractive Surgery
